# Prevalence of exclusive breastfeeding and barriers for its continuation up to six months in Kandy district, Sri Lanka

**DOI:** 10.1186/s13006-018-0180-y

**Published:** 2018-08-09

**Authors:** Himali Erandathie Ratnayake, Dhammica Rowel

**Affiliations:** 1 Epidemiology Unit, Colombo 10, Sri Lanka; 2 Family Health Bureau, Colombo 10, Sri Lanka

**Keywords:** Exclusive breastfeeding, Prevalence of exclusive breastfeeding, Barriers for exclusive breastfeeding

## Abstract

**Background:**

Since 2005, the national policy on breastfeeding in Sri Lanka is exclusive breastfeeding up to 6 months, as recommended by World Health Organization. The objective of this study was to assess the prevalence of exclusive breastfeeding and barriers for its’ continuation up to 6 months, in Kandy District, Sri Lanka.

**Methods:**

A clinic based cross-sectional study was conducted from August to November 2016, in six randomly selected Medical Officer of Health areas in the Kandy District. The sample was selected proportionate to the population of each Medical Officer of Health area and 354 mothers with infants aged 6 months, attending the child welfare clinics were recruited. Data were collected by an interviewer administered questionnaire using mother recall data since birth. A focus group discussion was conducted on 21 mothers who discontinued exclusive breastfeeding early. The infant taking only breast milk and no additional food, water, or other fluids with the exception of medicines and vitamins or mineral drops for the first 6 months was used as the definition of exclusive breastfeeding.

**Results:**

The prevalence of exclusive breastfeeding for 6 months was 50.8% (180/354) while the median duration was 6 months. Mother being employed (AOR 3.01; 95% CI 1.45, 6.29), mother’s poor knowledge on what she meant by exclusive breastfeeding (AOR 3.75; 95% CI 2.14, 6.54) and mother’s poor attitudes towards exclusive breastfeeding (AOR 2.98; 95% CI 1.76, 5.03) were independently associated with early cessation of exclusive breastfeeding. Unsupported environment in public places was not significantly associated with early cessation of exclusive breastfeeding. Focus group discussion revealed controversial health messages on exclusive breastfeeding delivered at different points of healthcare delivery, cultural practices which discouraged exclusive breastfeeding and difficulties in obtaining maternity leave as barriers for exclusive breastfeeding.

**Conclusions:**

The prevalence of exclusive breastfeeding up to 6 months was not satisfactory and there were barriers identified in healthcare system, family and work places towards exclusive breastfeeding. For further improvement in the prevalence of exclusive breastfeeding these issues need to be addressed and necessary changes in legislation implemented.

## Background

The World Health Organization (WHO) recommended exclusive breastfeeding (EBF) for 6 months in 2002 [1]. Sri Lanka extended the policy on duration of EBF from 4 to 6 months to 6 months in 2005.

According to 2016 Demographic and Health Survey (DHS) data in Sri Lanka, the prevalence of EBF of infants 0–6 months was 82%, while the EBF rate at 4–5 months was 64% [2]. As DHS calculated EBF by a 24-h dietary recall, it is possible that this rate be an over estimate [3, 4]. Furthermore, studies conducted in different districts in Sri Lanka reported different rates of exclusive breastfeeding. A cohort study conducted in 2008 in Naula MOH area using three methods; prospective data since birth, mother reported data and retrospective data based on an event calendar, revealed that prevalence of EBF up to 6 months was 23.9, 77.7 and 41.3% respectively [5]. A study conducted in Beruwala MOH area in 2006 found that the prevalence of EBF for 6 months was 15.5% [6], while according to a follow up study conducted in Gampaha district in 2010, the prevalence of EBF for 6 months was 71.3% [7]. The latter two studies used breastfeeding data since birth.

Most studies conducted in Sri Lanka reported an association between premature discontinuation of EBF and sociodemographic characteristics such as, ethnicity, education, occupation, age and birth order [6–8]. Studies to identify barriers within the health system, family, work place and public places, towards EBF are minimal in Sri Lankan setting. However, a substantial number of international studies disclose information regarding the above aspects [9–15].

The purpose of conducting this study was to assess the prevalence of EBF, using mother recall data since birth and to identify barriers within the health system, family, work place and public places towards EBF which were less well studied. The mother’s knowledge, attitudes and sociodemographic characteristics towards early cessation of EBF were also assessed.

## Methods

### Study setting and participants

A clinic based descriptive cross-sectional study was conducted from August to November 2016 in Kandy district, Sri Lanka, using both quantitative and qualitative data collection methods. Kandy district is situated in the Central Province of Sri Lanka and has a population of 1,375,382 according to census of population and housing 2012. The district has 23 Medical Officer of Health (MOH) areas where a Medical Officer of Health area refers to a small health administrative area in a district. Kandy consists of ethnic groups that speak two main languages; Sinhala and Tamil.

The study was conducted on mothers of children who have completed 6 months of age (age was checked with the Child Health Development Record), utilizing the immunization services at child welfare clinics for 6 months’ vaccination. Foster mothers, mothers having contraindications to breastfeed and mothers having a child with feeding difficulties were excluded. The sample size was calculated using confidence interval as 95%, precision level as 5%, and design effect as 1.2 [16]. The estimated prevalence of EBF up to 6 months was 77.7%, as reported by a study conducted in Naula MOH area in the Central Province, which used mother recall data at 9 months [5]. The total sample size of the quantitative study was 354.

For the focus group discussion (FGD), mothers who discontinued EBF early were selected. Saturation point was reached after conducting four focus group discussions with 21 mothers.

### Study sample

Six MOH areas were selected randomly out of the 23 MOH areas using a table of random numbers. The number of mothers selected from each MOH area was proportionate to the population of each MOH area (proportionate sampling). There were 10 to 18 clinic centres in one MOH area. The three data collectors visited three clinics each day. In instances where more than three clinics were held on same day, three clinics were randomly selected. Mothers were recruited for the study consecutively according to the registration order at the clinic, until the required number from each MOH area was achieved. In instances where a mother did not give consent to participate, the next mother was selected. A pilot study was conducted in a sample of 20 mothers attending child welfare clinic in another MOH area in the Kandy District, which was not included in the study proper.

Mothers for the FGD were selected randomly at the time of data collection for the quantitative component.

### Data collection

Quantitative data on sociodemographic factors, knowledge and attitudes on EBF and barriers for EBF in the health system, family, public places and work places were collected by an interviewer administered questionnaire. The data for quantitative study were collected by the principal investigator (PI), a retired medical officer (MO1) and a pre-intern medical officer (MO2). The data collectors were trained by the PI. Mother recall data from birth was used to gather information. Informed consent was obtained from participants after explaining the purpose of the study, participation method, advantages and disadvantages of participation.

Focus group discussions were conducted by the PI while the MO1 took down notes. A semi structured guide was used to interview the mothers who participated in the FGD. All mothers participated in the FGD were fluent in Sinhalese. One FGD lasted for about 30 to 40 min. The FGDs were not audio recorded due to the lack of facilities in many clinic settings. Mothers who participated in the discussion were coded with numbers to make information anonymous.

### Data measures and statistical analysis

Prevalence of EBF up to 6 months was described as a percentage and median duration of EBF was calculated. Mothers were asked two separate questions; “For how many months did you feed the child only with breast-milk?” and “Did you give water or any other preparation to the child during the above-mentioned period?” to gather information on prevalence of exclusive breastfeeding. The mother’s sociodemographic characteristics, knowledge, attitudes and identified barriers in the health system, family, public places and work place were described as frequencies and percentages. Mother’s knowledge on expressed breast milk was assessed by evaluating her knowledge on how to feed the baby when she was away, and her knowledge on methods and duration of storing expressed breast milk. There were 15 components in this section. Each correct response was scored ‘1’ and each incorrect response was scored ‘0’. Then each mother was given a mark out of 15. Median value was used as the cut off to develop a composite variable as, mothers having good knowledge and mothers having poor knowledge on expressed breast milk.

Association of independent variables were compared with dependent variable (continuation of EBF up to 6 months), using Chi square test at 95% significance level and odds ratio. Multivariate logistic regression was performed following bivariate logistic regression to determine independent predictors of premature discontinuation of exclusive. Quantitative data were analyzed using SPSS version 20 software.

Data collected from the FGDs were carefully analyzed by thematic areas with reference to context, frequency and extensiveness by the PI and a public health specialist in the field of Maternal and Child Health.

The WHO definition of exclusive breastfeeding: feeding the infant only with breast milk (including milk expressed or from a wet nurse) for the first 6 months of life, with the exception of oral rehydration solutions or drops/syrups of vitamins, minerals or medicines was used [17]. The mother’s attitudes and knowledge which had any negative impact on the continuation of EBF up to 6 months and any event that discouraged the continuation of EBF for 6 months, in the health system, family, public places or work places were considered as barriers for exclusive breastfeeding.

## Results

Six mothers (1.67%) did not consent to participate. The total number of mothers who participated in the study was 354. All mothers were fluent in conversational Sinhalese. Mean age of the study sample was 30.3 years (SD = 5.3) and the majority of the mothers were between 20 and 34 years (75.2%). Except for two, all mothers were educated at least up to grade 11 at school. Sociodemographic characteristics are summarized in Table 1.

**Table 1:** Distribution of the study sample by sociodemographic characteristics (*n* = 354)

Sociodemographic characteristics	*n*		%	
Age category				
≤ 19 years	11		3.1	
20–34 years	268		75.7	
≥ 35 years	75		21.2	
Ethnicity				
Sinhalese^a^	237		66.9	
Muslim ^b^	72		20.3	
Tamil ^b^	44		12.4	
Malay ^b^	1		0.3	
Education level				
Up to grade five ^c^	2		0.6	
Up to Ordinary Level ^c^	153		43.2	
Up to Advanced Level ^d^	172		48.6	
Degree/ Post graduate ^d^	27		7.6	
Occupation	Individual data (*n*)	Pooled data (*n*)	Individual data (%)	Pooled data (%)
Not employed ^e^	291	301	82.2	84.5
Self-employed ^e^	10		2.3	
Teacher ^f^	13	53	3.7	15.5
Clerical ^f^	27		2.8	
Labourer ^f^	8		7.6	
Executive ^f^	5		1.4	
Household Income				
≤ 2 5000	127		35.9	
25,001-50,000	131		37	
≥ 50,001	96		27.1	
Birth order of the child				
First	124		35	
^g^Second	128		36.2	
^g^Third	83		23.4	
^g^Fourth	19		5.4	
Total	354		100	

In further analysis data were combined as follows

^a^Sinhalese, ^b^Other, ^c^Up to grade 11 or less, ^d^Above grade 11, ^e^Unemployed, ^f^Employed & ^g^Birth order second or above

The prevalence of EBF up to 6 months was 50.8% (180/354) and EBF up to 5 months or more was 81.3%. Median duration of EBF was 6 months. Table 2 illustrates the rate of EBF continued only up to a given month.

**Table 2:** Distribution of rate of EBF continued only up to a given month (*n* = 354)

Duration of EBF	Individual data		Pooled data		
	*n*	%		*n*	%
^a^Only up to 1 month	5	1.4	5 months or less	174	49.2
^a^Only up to 2 months	2	0.6			
^a^Only up to 3 months	10	2.8			
^a^Only up to 4 months	49	13.8			
^a^Only up to 5 months	108	30.5			
Only up to 6 months	180	50.8	Up to 6 months	180	50.8
Total	354	100		354	100

^a^In further analysis these were combined as “EBF for less than 6 months”

The main reason for early cessation of EBF was the mother thinking that breast milk only was not enough for the baby (52.9%; 92/174). Items given to the baby before 6 months were water (91.4%; 159/174), fruit juices (83.9%; 146/174), mashed rice (71.3%; 124/174) and formula milk (16.1%; 28/174). The majority of mothers (98.9%, 350/354) knew that the current recommendation of duration of EBF was 6 months. Only 27.7% of mothers (98/354) could correctly define the term ‘exclusive breastfeeding’.

The assessment of the mothers’ knowledge on composition and benefits of breast milk is illustrated in Table 3.

**Table 3:** Distribution of mothers’ knowledge regarding composition and benefits of breast milk (*n* = 354)

^a^Response	Correct response	*n*	%
Breast milk gives immunity to the baby	True	352	99.4
Breast milk gives enough water for the baby	True	264	74.6
Breast milk helps brain development of the baby	True	352	99.4
Breastfed babies gain weight slowly	False	293	82.8
Breastfeeding is the most cost-effective way to protect baby from diarrhoeal diseases	True	335	94.6
Breastfeeding protects mother from breast cancers	True	316	89.3
Exclusive breastfeeding helps mother in preventing getting pregnant too early	True	80	22.6
Breastfeeding affects mother’s health badly	False	277	78.2
Breastfeeding helps to build up a good bond between mother and baby	True	353	99.7
Formula milk has similar benefits as breast milk	False	346	97.7

^a^Multiple response variables

The majority of mothers (92.4%, 327/354) knew that the baby should be given expressed breast milk when mother was away, while 62.4% (221/354) believed that giving formula milk was an option. The percentage of mothers who knew breast milk can be expressed and kept stored was 68.4% (242/354). Out of them 65.3% (231/242) knew it could be stored at room temperature while 48% (170/242) and 12.1% (43/242) knew it could be stored in a refrigerator and freezer compartments.

Forty-three (12.1%) mothers said they were advised by a healthcare worker to start feeds other than breast milk during the first 6 months. Of these mothers, 28 were advised by a doctor. Only 29 mothers (8.2%) started formula feeds within the first 6 months. Of these, 20 (69%) were on prescription of a healthcare worker. The majority of mothers (96%, 340/354) found it was not difficult to get help from a healthcare worker when they needed assistance in breastfeeding. The majority of babies (94.6%, 335/354) were breastfed within 1 h of delivery. Of them 77.7% (275/354) mothers believed that their babies were not allowed to complete the first breastfeed.

We found that 50.6% of mothers were advised by family members to cease EBF early. Most commonly the family member was their mother-in-law (55.9%) or their mother (44.7%), while, 29.1% said that their husbands influenced them negatively. The majority of the mothers (81.1%, 287/354) had good family support. Half of the mothers perceived that their family members had encouraging attitudes towards breastfeeding.

Of the 53 working mothers, only 58.5% (31/53) obtained maternity leave for 6 months. Nearly half of the mothers (47.2%, 25/53) reported it was difficult to obtain leave due to work place regulations and inadequate staff. Of the 22 mothers who left for work before 6 months, 20 did not practice expressing breast milk at work place (Table 4).

**Table 4:** Reasons given by mothers for not expressing breast milk at work place (*n* = 20)

Reason for not expressing	*n*	%
No suitable place	10	50
No place to store	3	15
Busy with work	2	10
I didn’t want to	4	20
I didn’t know	1	5
Total	20	100

Twenty of the twenty-two mothers said that their superiors and work mates at work place had neutral or discouraging attitudes towards expressing breast milk at work place.

Difficulties faced by mothers when feeding their baby in public places is illustrated in Table 5.

**Table 5:** Distribution of method of feeding and difficulties mothers faced when breastfeeding in public places (*n* = 354)

Variables	*n*	%
Type of feeding given to baby		
Breastfeeding or expressed breast milk mostly	231	65
Feeds other than breast milk mostly	123	35
Feeling embarrassed to breastfeed in public places		
Yes	313	89
No	26	7
Breastfed inside the private vehicle	15	4
Availability of feeding corners in public places mothers visited		
Yes, most of the times	242	69
Sometimes found	97	27
Never	15	4
Total	354	100

Multivariable logistic regression analyses indicated that maternal employment status, mothers’ knowledge on what she meant by EBF and mothers’ attitudes towards exclusive breastfeeding were significant predictors of duration of exclusive breastfeeding. The adjusted odds of employed mothers discontinuing exclusive breastfeeding early was 3.4 times the odds of unemployed mothers (AOR 3.4; 95% CI 1.6, 7.2). Mothers poor knowledge on what she meant by exclusive breastfeeding (AOR 3.8; 95% CI 2.1, 6.7) and her poor attitudes towards exclusive breastfeeding (AOR 2.8; 95% CI 1.7, 4.7) were also significantly associated with premature discontinuation of exclusive breastfeeding (Table 6).

**Table 6:** Multivariate logistic regression for potential predictors of premature discontinuation of exclusive breastfeeding

Variable	Unadjusted odds ratio (95% CI)	Adjusted odds ratio (95% CI)
Mother’s age		
≤ 19 years	1	
20–34 years	1.6 (0.3,7.4)	
≥ 35 years	2.9 (0.5,14.9)	
Ethnicity		
Sinhalese	1	
Other	0.9 (0.6,1.5)	
Level of education		
Up to grade 11 or less	1	
Above grade 11	0.6 (0.4,1.04)	
Mother’s occupation		
Unemployed	1	1
Employed	4.3 (2.1,8.5)	3.4 (1.6,7.2)
Birth order of the child		
Second or above	1	1
First child	1.8 (1.1,2.8)	1.4 (0.9,2.4)
Mother’s knowledge on what is meant by EBF		
Good knowledge	1	1
Poor knowledge	2.9 (1.9,4.5)	3.8 (2.1,6.7)
Mothers’ knowledge regarding composition and benefits of breast milk		
Good knowledge	1	
Poor knowledge	1.5 (0.9,2.2)	
Mothers’ attitudes towards breastfeeding		
Good attitudes	1	1
Poor attitudes	1.6 (1.02,2.6)	2.8 (1.7,4.7)
Difficulty in getting help from healthcare workers when mother had problems regarding breastfeeding		
Not difficult	1	1
It was difficult	3.9 (1.1,14.5)	3.7 (0.8,15.7)
Timely initiation of breastfeeding		
Yes	1	
No	1.5 (0.6,5)	
Family members influence to give feeds other than breast milk		
Yes	1.6 (0.9,2.5)	
No	1	
Support from family members		
Good support	1	1
Poor support	1.7 (1.01,2.9)	1.8 (0.9,3.3)
Difficulty in getting maternity leave (*n* = 53)		
Yes	3.5 (0.8,14.7)	
No	1	
Feeling embarrassed to breastfeed in public places		
Yes	1.01 (0.5,1.9)	
No	1	
Availability of feeding corners in public places the mothers visited		
Yes, most of the time	1	
Sometimes	0.6 (0.2,1.9)	
Never	1.0 (0.4,2.9)	

### Focus group discussion

The ages of 21 mothers ranged from 19 to 38 years. The majority of the mothers were Sinhalese and Buddhists (*n* = 15, 71.4%) while five (23.8%) were Muslims and one (4.8%) was Tamil. Only three mothers (14.3%) were employed.

All mothers knew the recommended duration of exclusive breastfeeding. Almost all mothers revealed that health education sessions on breastfeeding were conducted at MOH clinics and were very useful. They all agreed that their Public Health Midwives were friendly and helpful. Mothers said that the physicians did not provide much encouragement for breastfeeding.

“The importance of EBF for six months was a frequent message at antenatal clinics at MOH but, my obstetrician never talked about breastfeeding throughout the nine months (29 years).”

“I started complementary feeding at four months as I did with my elder son. The paediatrician knew it but, he didn’t say anything (35 years).”

One mother (31 years) who delivered the baby in a private hospital explained her negative experience by saying;

“The staff was not at all helpful and the nurses did not help to breastfeed the baby. They even suggested on giving a formula feed as the attachment was not good.”

Mothers listened to the advice of family members and relatives in deciding when to start complementary feeding. Eight mothers said their mothers and mothers-in-law insisted on giving water, fruit juice, and coriander water to the baby. All Sinhalese mothers gave “Ratha Kalkaya” (an Ayurwedic product) to their babies as told by their mothers and mothers-in-law. One mother said that her mother put small amounts of water from the water basin to the baby’s mouth while bathing the baby, which was a ritual.

Mothers encountered difficulties when training the caregiver on feeding the baby with expressed breast milk. One mother said she kept expressed breast milk at home but kept formula milk also ready, since her mother once spilled the expressed breast milk.

One mother (34 years), a laborer in a private company, described the difficulties in getting maternity leave by saying;

“We do not have such leave called ‘maternity leave’ and if we take leave we are not paid since we are paid on a daily basis.”

Employed mothers described the unsupportive environment at work places for nursing mothers. One mother (31 years) said that there were CCTV (closed circuit television) cameras all over, so she didn’t want to express breast milk at the work place. Another said she had to go to the toilet to express breast milk.

All mothers used to go out of home by their own vehicle or by a known vehicle where they could feed the baby and they all agreed that they felt embarrassed to feed the baby in public places. The majority did not go out during the first 6 months, other than for vaccinating the child.

“It was really embarrassing to breastfeed the baby inside a bus, because usually buses are crowded and people sometimes stare at nursing mothers (28 years).”

## Discussion

According to this study only half of the population (50.8%) exclusively breastfed up to 6 months. As DHS gathered information by a 24-h dietary recall, it was difficult to compare the current study with DHS data. Contrary to the findings in the present study, previously conducted studies in Sri Lanka reported better EBF rates. According to these studies EBF rates at 6 months were 71.3% in 2007 [7], 72% in 2009 [8] and 62.2% in 2010 [18]. The probable reason for this could be the differences in breastfeeding definitions, varying study settings and varying methodologies. It is important to explore the reasons for discontinuing EBF at 5 months as it was found that a large proportion of mothers (30%) discontinued EBF at 5 months. A great proportion of mothers (52.9%) believed that breast milk only was not enough for the baby, as found by previous studies [8, 19, 20]. The probable reason for this could be a deficiency in health education given to the mothers.

Mother being employed had a threefold risk of discontinuing EBF early compared to mothers not employed, and could be due to the discrepancy in maternity leave benefit in different work settings. In Sri Lanka, private companies allow a shorter maternity leave compared to government institutions. A revision in the legislation is needed in Sri Lanka in this regard. However, contrary to this finding, a study done in Beruwala reported a positive correlation of EBF with maternal employment where the author stated it could be due to the small number (7.3%, 16/203) of working mothers included in the sample [6]. There were nearly 60% mothers who had the impression that, giving a sip of water or fruit juice, while predominantly breastfeeding was the recommended practice of exclusive breastfeeding. Individual counseling to mothers by public health staff would help in correcting these misconceptions. Mothers’ knowledge on composition and benefits of breast milk was satisfactory as found by similar studies [8, 21, 22].

It seemed that a large number of mothers believed that giving formula milk was an option when mother was away. Lack of knowledge on expressed breast milk could be the probable reason for this. Health messages on expressing breast milk can be displayed in clinics or can be included in to mother’s Antenatal Record or Child Health Development Record.

It was found that the mother’s negative attitudes regarding breastfeeding had a significant association towards early cessation of exclusive breastfeeding. There were similar findings reported from other countries as well [23–25] which needs further exploration.

It was evident that healthcare workers advised the premature initiation of complementary feeding. This seemed to be a problem even in developed countries like United States [11] and England [13]. A mechanism to update the knowledge of healthcare workers would help to overcome this situation. However, the public health midwives (PHMs) were appreciated by mothers in this study as well as previously conducted studies [8], which is a positive remark of the health system in Sri Lanka.

The initiation of breastfeeding within the first hour of delivery was commendable (94.6%) and similar to DHS-2016 results (90%) [2]. This was a 10% increase compared to the findings of DHS 2006–2007, 10 years previously [26]. The majority of mothers perceived that babies were not allowed to complete the first feed. This finding would have been more accurately interpreted if this was observed by investigators.

Apart from their mothers and mothers-in law, a reasonable proportion of mothers reported negative influences from their husbands towards exclusive breastfeeding. Previously conducted studies in Sri Lanka also reported similar findings [8, 27]. Studies conducted outside the Asian context too reported similar findings [12, 14, 28, 29]. Family members’ participation at health education sessions should be increased in this regard. At home visits, PHMM should be encouraged to pay special attention to the family members who did not participate at health education sessions.

Regulating the existing labour laws is a timely need as nearly half of the working population revealed that they encountered difficulties in getting the approval for obtaining maternity leave. Working mothers should be empowered to utilize maternity leave benefit even in unsupportive environments. A separate place for expressing breast milk at every work place also should be implemented. Two studies done in Australia also revealed difficulties faced by working mothers [15, 30].

Previously conducted studies reported that unavailability of a proper place to breastfeed in public places forced mothers to shift to alternative feedings [31–33]. Incorporating breastfeeding friendly settings in city development plans would help to overcome this, as most mothers stated that they were embarrassed to breastfeed in public places.

The qualitative component of this study identified cultural practices pertaining to different ethnic groups and misleading information delivered by healthcare workers. Qualitative studies on breastfeeding were rare in Sri Lankan setting. However similar findings were elicited from qualitative studies conducted in other countries [14, 31, 34].

There were several limitations of this study. Prospective method was not used to conduct the quantitative study due to time constraints, though it was the ideal. The study did not assess ‘any breastfeeding’ to see whether there was a drop in ‘any breastfeeding’ with age. The percentage of working mothers was low compared to national figures. This could be due to working mothers getting services from the private sector. This study excluded mothers who utilized the private sector for vaccination. The results would have been more accurate if these mothers also were included. ‘Breastfeeding’ and ‘feeding the child with expressed breast milk’ when in public places was not separately analysed which could have elicited more information. The qualitative study group did not include a sufficient number of mothers from some ethnic categories to do a better analysis. Audio taping which would have improved analysis, was not done due to lack of facilities in clinic settings.

## Conclusions

The prevalence of exclusive breastfeeding was not satisfactory. The fathers, other healthcare workers, employers, staff in work places and the community need to be made aware of importance of exclusive breastfeeding. It is time to change the legislation in order to provide 6 months maternity leave for all working mothers, to make all work places breastfeeding friendly and to make feeding places available at all public places.

## Abbreviations

DHS: Demographic and Health Survey
EBF: Exclusive breastfeeding
G.C.E. A/L: General Certificate of Education (Advanced Level)
G.C.E. O/L: General Certificate of Education (Ordinary Level)
HCW: Healthcare Worker
MO: Medical Officer
MOH: Medical Officer of Health
OR: Odds Ratio
PHM: Public Health Midwife
PHMM: Public Health Midwives
SPSS: Statistical Package for the Social Sciences
WHO: World Health Organization
